# Comparative mortality in pituitary adenomas subtypes: a tertiary referral center study

**DOI:** 10.1007/s12020-024-04073-y

**Published:** 2024-10-19

**Authors:** Iustin Daniel Toma, Dan Alexandru Niculescu, Simona Andreea Găloiu, Raluca Alexandra Trifănescu, Cătălina Poiană

**Affiliations:** 1 Department of Endocrinology, Carol Davila University of Medicine and Pharmacy, Bucharest, Romania; 2 First Endocrinology Department, C. I. Parhon National Institute of Endocrinology, Bucharest, Romania

**Keywords:** Mortality, Acromegaly, Cushing’s Disease, Prolactinoma, Non-functioning pituitary adenoma

## Abstract

**Purpose:**

Mortality in pituitary adenomas (PAs) has been extensively compared to general population. However, direct comparisons between PA subtypes are scarce. We aimed to compare mortality in various subtypes of PA (acromegaly, Cushing’s disease (CD), macroprolactinomas and non-functioning pituitary macroadenomas (MacroNFPA)), within a single referral center.

**Methods:**

We retrospectively analyzed mortality and survival time in all 962 PAs admitted in our department between 2011 and 2023: acromegaly (*n* = 306), CD (*n* = 69), macroprolactinoma (*n* = 168) and MacroNFPA (*n* = 419).

**Results:**

Median follow-up was 10.2 (5.2, 15.2) years. The overall survival probability was 90.9% and 78.1% after 10 and 20 years respectively with age at diagnosis as the only significant predictor. There were no significant differences in survival probability between various PA subtypes in the whole cohort. In patients over 45 years of age at diagnosis there was a significant difference in survival probability between the four groups (*p* = 0.01) in the first 15 years of follow-up. In head-to-head comparisons CD had a significantly higher mortality risk than patients with acromegaly (HR 3.38 [CI 95% 1.07 to 10.60]) even after adjusting for age and sex.

**Conclusion:**

Patients diagnosed with CD after 45 years of age have a significantly lower survival probability than other PA subtypes in the first 15 years of follow-up. All other PA subtypes had a similar survival probability after adjusting for age and sex. Due to advances in medical treatment of hormone secreting tumors, mortality in patients with PAs might increasingly depend more on tumor mass than on hormonal hypersecretion.

## Introduction

Pituitary adenomas (PAs) are the most common pituitary tumors and, in the vast majority of cases, have a clinically benign evolution. Moreover, PA represent approximately 15.5% of all brain and central nervous system tumors [1]. The prevalence differs widely due to various diagnostic methods, from 22.5% in autopsy studies back in 1936 [2] to 14.4% in other systematic reviews [3]. A nation-wide study, conducted by Agustsson et al. [4] showed that PAs have increasing rates of prevalence and incidence, mostly due to ease of access to modern imaging techniques.

Mortality in PAs depends on the histological subtype. Acromegaly was historically associated with an increased mortality but recent meta-analyses showed that due to new diagnostic methods and more effective therapies mortality steadily decreased in the last 10–15 years and the life expectancy is now approaching that of the general population with a SMR of 1.35 (95% CI 0.99–1.85) [5] or 1.2 (95% CI 1.0–1.4) [6]. However, some recent studies report a significantly increased SMR for women [7, 8]. Patients with Cushing’s disease (CD) have an increased overall mortality with a standardized mortality ratio (SMR) of 3.22 (95% CI 1.7–5.6) [9]. However, mortality was significantly higher only in patients with active disease (SMR 4.99, 95%CI [2.15; 9.83]), but was similar to that of the general population in those in remission (SMR 1.66, 95%CI [0.34; 4.85]) [9]. Mortality rates were much less studied in prolactinomas. The only recent paper that reported SMR in prolactinomas at a nation-level found not significant differences (SMR 1.1 [95% CI 0.93–1.3]) [10]. Mortality is still increased in patients with non-functioning pituitary adenomas (NFPA). A recent meta-analysis [11] reported a SMR of 1.57 (95% CI 1.20–1.99). This excess of mortality was observed particularly in women and in those diagnosed before 40 years of age but, surprisingly, was not dependent on the presence of hypopituitarism [11].

In contrast to the many studies that assessed mortality in PAs and general population, direct comparisons between PA subtypes are scarce. Oh et al. [10] conducted a nation-wide study in Korea and observed a higher SMR in NFPA and CD than in prolactinoma or acromegaly. However, no direct comparisons were made. Moreover, it is likely that prolactinomas included microadenomas and NFPA, particularly those without hypopituitarism, included a large proportion of pituitary incidentalomas. Excluding the study of Oh [10], comparing mortality from different studies is not reliable as there are important genetic or health care differences between populations and significantly different methodologies.

Our aim was to directly compare mortality rates in different subtypes of pituitary adenomas (acromegaly, Cushing’s, non-functioning pituitary macroadenomas and macroprolactinomas), in a single large referral center.

## Methods

### Patients

This is a retrospective study conducted within a tertiary referral endocrine center in Bucharest, Romania. We analyzed the charts of all patients that had been admitted in our department between 1st of January 2011 and 1st of April 2023 with at least one of the following ICD-10 codes: [D35.2]—Benign pituitary tumor, [C75.1]—Pituitary carcinoma, [D44.3] - Pituitary tumor with unpredictable and unknown evolution, [E22.0]—Acromegaly, [E22.1]—Hyperprolactinemia, [E24.0]—Pituitary Cushing’s disease, [E22.8]—Other hypersecretions of the pituitary gland, [E22.9]—Pituitary gland hypersecretion, unspecified, as primary or as secondary diagnosis. Each chart was thoroughly examined and only patients with pituitary adenomas (acromegaly, Cushing’s disease, macroprolactinoma, non-functioning pituitary macroadenoma) were included in the study. “Adenoma” was preferred instead of “PitNET” as this is a retrospective study with a prolonged follow-up period, during which the majority of the included tumors were classified as “adenomas” in terms of diagnosis, treatment, prognosis and overall clinical management by the treating physicians. Moreover, our study focuses primarily on clinical diagnosis and disease progression, rather than on pathological classification. The positive diagnosis was made according to the international guidelines available at that time. A macroprolactinoma was diagnosed based on a pituitary macroadenoma visible on CT/MRI scans and a serum prolactin level exceeding 200 ng/mL. Acromegaly was diagnosed based on a minim serum growth hormone after 75 g oral glucose load >1 ng/mL (before 2022) or >0.4 ng/mL (after 2022) and an IGF-1 serum level above the upper limit of normal. Cushing disease was diagnosed in individuals with cortisol levels exceeding 50 nmol/L after a low dose dexamethasone suppression test in the presence of normal or high ACTH and a pituitary adenoma on CT/MTI scans and no clinical, biochemical or imaging evidence of ectopic ACTH production. A non-functioning pituitary macroadenoma was diagnosed in patients with pituitary tumors at least 10 mm on CT/MRI scans and no signs of hormonal hypersecretion. MicroNFPAs and microprolactinomas were excluded due to very low mortality and the high risk of false positive diagnosis. Thyrotrophinomas, clinical gonadotropinomas, pituitary carcinomas and patients with multiple endocrine neoplasia were excluded due to the very low number of cases. Also, non-adenomatous sellar masses like craniopharyngiomas or meningiomas were excluded due to the low number of cases and different clinical behavior or treatment. The study was approved by the Ethics committee from “C.I. Parhon” National Institute of Endocrinology, Bucharest, Romania (No 9/09.02.2023).

The following data were recorded: sex, age at diagnosis, maximum tumoral diameter and disease duration. Readmissions, inaccurate code reporting and patients with insufficient data about their illness were excluded. Survival status (dead or alive) at the end of study (01.04.2023) and date of death were provided by The General Directorate for Persons Record (http://depabd.mai.gov.ro/) which is the governmental entity responsible for maintaining updated records and managing databases related to individuals in Romania. The duration of illness was determined from the first day of confirmed diagnosis.

### Statistics

All continuous variables had a non-parametric distribution by the Kolmogorov-Smirnov test and are presented as median (25, 75 percentile). Differences between all 4 groups of patients (acromegaly, Cushing, macroprolactinoma and MacroNFPA) were assessed using the Kruskal-Wallis test with no post-hoc testing. Time to event variables were analyzed using the Kaplan-Meier method using the date of diagnosis as the starting point. A stepwise Cox proportional-hazards regression was used to calculate mortality hazard ratio (HR) after adjustment for the age at diagnosis and sex. As age was the most significant predictor of death, in order to construct meaningful Kaplan-Meier curves we used a cut-off for age at diagnosis that best approximated the median in all four subgroups, namely 45 years of age. As such, Kaplan-Meier curves were constructed for those under of above 45 years of age at diagnosis and hazard ratios were calculated for limited follow-up, namely 40 years for those under the age of 45 and 15 years for those above the age of 45.

## Results

**Fig. 1:**
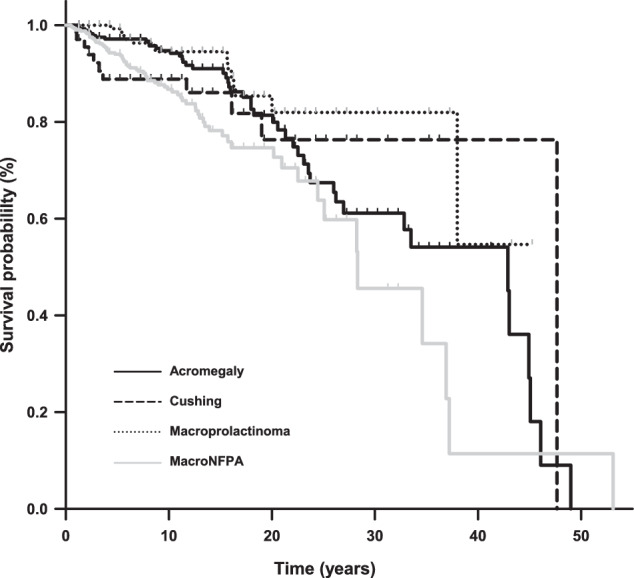
Survival probability in patients with acromegaly (solid black line), Cushing’s disease (dashed line), prolactinoma (dotted line) and MacroNFPA (solid gray line)

Subjects’ characteristics can be found in Table 1. There were clinically significant differences in the age at diagnosis and sex distribution between the 4 groups of patients. Also, there were statistically significant differences in the year of diagnosis and duration of follow-up but of minimal clinical significance. Figure 1 shows the Kaplan-Meier curves for the whole follow-up period in the 4 types of pituitary tumors. Supplementary Table 1 shows the number of deaths and survival probabilities at 5, 10, 20, 30 and 40 years of follow-up in the whole cohort and in those under or above 45 years of age at diagnosis.

**Table 1:** Subjects’ characteristics

	Total (*n* = 962)	Acromegaly (*n* = 306)	Cushing (*n* = 69)	Macroprolactinoma (*n* = 168)	MacroNFPA (*n* = 419)	*p* value
Age at diagnosis, years	48.2 (35.9, 59.7)	44.4 (36.0, 54.4)	43.1 (31.9, 55.9)	36.2 (27.0, 52.1)	54.8 (41.9, 64.1)	<0.001
Males, n (%)	413 (42.9%)	100 (32.6%)	16 (23.1%)	96 (57.1%)	201 (47.9%)	<0.001
Macroadenomas, n (%)	860 (89.3%)	253 (82.6%)	20 (28.9%)	168 (100%)	419 (100%)	NA
Year of diagnosis	2013 (2007, 2017)	2012 (2005, 2017)	2013 (2006, 2019)	2011 (2005, 2016)	2014 (2010, 2018)	<0.001
Follow-up, years	10.2 (5.2, 15.2)	11.2 (5.2, 16.2)	10.2 (4.0, 17.2)	11.2 (6.3, 17.2)	8.2 (5.2, 13.2)	<0.001

Data are presented as median (25, 75 percentile) or as number (percent)

**Fig. 2:**
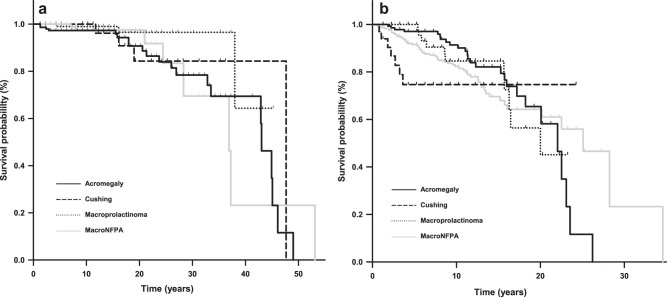
Survival probability in patients with acromegaly (solid black line), Cushing’s disease (dashed line), prolactinoma (dotted line) and MacroNFPA (solid gray line) under 45 years of age at diagnosis (**a**) and over 45 years of age at diagnosis (**b**)

The stepwise Cox proportional-hazards regression retained age at diagnosis as the only significant predictor for mortality in the model with a hazard ratio of 1.097 for each additional year. Tumor type and sex, both as categorical variables, were not retained in the model. In patients under 45 years of age at diagnosis there were no significant differences in survival probability between any of the tumor types (Fig. 2a). In patients over 45 years of age at diagnosis there was a significant difference between the four groups (p value at log rank test = 0.01) in the first 15 years of follow-up (Fig. 2b). Head-to-head comparisons in patients over 45 years of age at diagnosis showed that Cushing disease and MacroNFPA had a significantly higher mortality risk than patients with acromegaly with a HR of 3.38 (CI 95% 1.07–10.60) and 1.93 (CI 95% 1.17–3.17) in the first 15 years of follow-up. The increase in mortality associated with CD vs. acromegaly remained significant after adjusting for age at diagnosis and sex in the Cox model. There were no significant differences in mortality between macroprolactinomas and the other tumor types.

## Discussion

Our study is the first to directly compare mortality between various subtypes of pituitary tumors, in a single large tertiary center. Only clinically relevant pituitary tumors (acromegaly, Cushing, macroprolactinoma and MacroNFPA) were included. Age at diagnosis and sex were considered as covariables.

We demonstrated an overall survival probability of 90.9% in the first 10 years after diagnosis. All tumor subtypes had a 10-year survival probability above 85%, ranging from 86.8% in MacroNFPA to 94.7% in acromegaly. Cox proportional-hazards analysis of the whole cohort showed that only age at diagnosis was significantly associated with an increased risk of death with a HR of 1.097 for each additional year. In patients over 45 years of age at diagnosis, CD and MacroNFPA had a significantly higher mortality risk than patients with acromegaly with a HR of 3.38 (CI 95% 1.07 to 10.60) and 1.93 (CI 95% 1.17–3.17) respectively. There were no significant differences in mortality between macroprolactinomas and the other tumor types regardless of age.

Recent studies that reported overall survival in acromegaly showed a 10-year probability of survival around 90% [8, 10, 12], similar to our study. As expected, survival was higher in patients with controlled acromegaly, usually above 95% for the first 10 years of follow-up [8, 12, 13]. It is important to note that the last report from our center showed an overall control rate in patients with acromegaly of 52% based on IGF-1 measurement [14]. Survival probability was significantly lower in the first 5 years after diagnosis of CD (88.9%) compared to other PA (97.2% in acromegaly, 99.3% in macroprolactinoma and 94.0% in MacroNFPA) but beginning with 10th year of follow-up the survival probability was similar or even higher than in the other PAs. These results are in line with those reported by Mondin et al. [9] who demonstrated a rapid decline in survival probability in the first 5–10 years after diagnosis, particularly in those with persistent disease. Ten years after diagnosis the survival probability was about 95% in those in remission or 85% in those with persistent disease [9] and 90% in the entire population [10] as compared with 88.9% in our entire cohort. Also, the only study that reported the survival probability in prolactinomas [10] found similar results to our study, namely a 10-year probability above 95% compared to 94.5% in our cohort. No data beyond this time point are available [10]. In patients with MacroNFPA we found a 5- and 10-year survival probability of 94.0% and 86.8% respectively. This is in accordance with published studies that reported a survival probability around 93 and 83% respectively in patients with NFPA and hypopituitarism [10] or survival rates of 90 and 79% respectively in a nationwide study [15]. Only 53% of patients from the last study were treated by surgery or radiotherapy.

When comparing survival probability between PA subtypes in the short term we found that CD is associated with the highest mortality. This is due exclusively to those diagnosed above 45 years of age Survival probability of CD remained significantly lower than that of acromegaly for the first 15 years of follow-up even after adjusting for age at diagnosis and sex. This is in accordance with a nationwide Korean study that reported a significantly increased SMR in those over but not in those under 40 years of age [10]. Also, Mondin [9] demonstrated an increased mortality in those diagnosed over 45 years of age compared with those diagnosed at a younger age. Interestingly, a study on 81 women with CD showed a significantly higher urinary free cortisol and ACTH in those diagnosed over 65 years of age as compared with those under 45 years [16]. However, if this increased mortality is due to a more active disease or pre-existent comorbidities is currently unknown. Acromegaly was found to have a similar survival probability to MacroNFPA after adjusting for age and sex. This might be due to increasing efficacy of medical treatment [5] or to better management of metabolic and cardiovascular comorbidities. As expected, macroprolactinomas demonstrated the highest survival probability of all PAs, 94.5% at 10 years and 82% and 30 years. This is in accordance with the high efficacy of dopamine agonists in the treatment of prolactinomas with largest studies reporting hypersecretion and tumor response rates to DA of 77% and 75–95% respectively [17, 18]. However, in our study this low mortality is mainly due to younger age at diagnosis, 18 years less than in MacroNFPA, and 8 years less than in acromegaly as these differences in mortality are no longer significant after adjusting for age and sex. We have to note that it is possible these differences could remain statistically significant if microprolactinomas would have been included in the study. Oh et al. [10] also found prolactinomas to have the highest survival probability but no statistical testing was employed. They reported a median age at diagnosis of 38 in prolactinomas vs. 51 in NFPA and 45 in acromegaly [10]. Studies on very large databases also showed that age is the most important predictor of survival in pituitary adenomas [19].

The only study [10] that directly compared mortality in PA subtypes (nationwide) showed that patients with prolactinomas had the highest and NFPA with hypopituitarism the lowest survival probability with acromegaly and CD in between. However, the differences in survival probability were not statistically tested and there was no adjustment for age or sex. Moreover, the median follow-up period was 5.3 and the maximum follow-up was limited to 10 years. To our knowledge there are no other studies to directly compare survival probability or SMR in PA subtypes. Our study proposes a significantly longer follow-up period and adjusts for the age of diagnosis and sex as there are clinical and statistically significant differences between subgroups.

All these findings show that PAs, with the notable exception of CD, have a similar survival probability after adjusting for age and sex. The first important conclusion is that PAs cancel the survival advantage of women that is evident in the general population. This finding was also emerging from numerous other studies that showed that in acromegaly or NFPA women but not men have a statistically increased SMR [20, 21]. Future studies should investigate whether PAs only increase the mortality in women or simultaneously decrease the mortality in men by a possible mild untreated hypocortisolism and hypogonadism. Of note, some studies that evaluated survival in all pituitary adenomas regardless of the secretory type showed that women have a better survival compared to men [19]. Secondly, it is tempting to suggest that PAs associated mortality is dependent more on the tumor mass and its compressive effects than on hormonal hypersecretion. This should not be an unexpected finding as latest studies report an overall hypersecretion control rate of 82% in long term in acromegaly [22] and 75–95% in macroprolactinomas [18] as a result of improved surgery and medical treatments.

An important limitation of our study is the relatively short follow-up. Although the median period of follow-up was 10.2 years, 25% of patients had been followed for less than 5.2 years and this might negatively impact mortality, particularly in younger patients. However, most published studies have a similar follow-up period [8, 10]. Also, another limitation is the relatively low number of patients with CD, a feature characteristic of single center studies. Our study has no data on the presence or severity of hypopituitarism or on the controlled/not controlled status of patients with secreting pituitary adenomas (acromegaly, macroprolactinoma, Cushing’s disease). Although important in studies involving only one type of tumor, these variables would lead to a significant increase in the number of subgroups and loss of statistical power. Also, tumor volume could be considered as a covariate. However, there is an important heterogeneity of imaging methods and accuracy ranging from unenhanced axial computed tomography 30 years ago to contrast-enhanced pituitary MRI in present days. Moreover, various diameters are reported while tumor volume is available in a minority of cases. Given all these inconsistencies we preferred not to include tumor diameter in our analysis. Also, all prolactinomas and non-functioning pituitary adenomas were macroadenomas by inclusion criteria while 82.6% and 28.9% of acromegaly and Cushing’s disease respectively were macroadenomas. Tumor volume distribution is roughly balanced between PA subtypes and its impact is less important in the way our analysis was constructed.

The main advantage of our study is the monocentric approach that limits the heterogeneity of diagnostic and therapeutic protocols and surgical performance. Also, we were able to include in the study only clinically significant tumors and to exclude microprolactinomas and pituitary incidentalomas that do not impact mortality but are difficult to exclude from insurance-claim based studies.

In conclusion, we showed that patients with CD diagnosed over 45 years of age have a significantly lower survival probability than other PA subtypes in the first 15 years of follow-up. In all PAs age at diagnosis was the most important prognostic factor for mortality. Apart from this, all PA subtypes (acromegaly, Cushing’s, macroprolactinomas and MacroNFPA) had a similar survival probability after adjusting for age and sex, both in the short and long term. It is tempting to suggest that mortality in acromegaly, macroprolactinomas and MacroNFPA could be dependent more on the tumor mass than on hormonal hypersecretion.

## Supplementary information

Supplementary Information

## Data Availability

No datasets were generated or analysed during the current study.
