# Supplementary material for: Comparative mortality in pituitary adenomas subtypes: a tertiary referral center study
Source: Endocrine. 2024 Oct 19;87(2):782–7. doi: 10.1007/s12020-024-04073-y (PMC11811481; doi:10.1007/s12020-024-04073-y)
Supplement: Supplementary file 1 — Supplementary Information [file 12020_2024_4073_MOESM1_ESM.docx]

| Supplementary Table 1. Number of deaths and survival probabilities by tumor type and follow-up period | | | | | | | | | | | |
| --- | --- | --- | --- | --- | --- | --- | --- | --- | --- | --- | --- |
|  | Total  (n=962) | | Acromegaly  (n=306) | | Cushing  (n=69) | | Macroprolactinoma  (n=168) | | MacroNFPA  (n=419) | | p-value |
| Follow-up  (years) by the age at diagnosis | Deaths  (n) | Surviving probability (%) | Deaths  (n) | Surviving probability (%) | Deaths  (n) | Surviving probability (%) | Deaths  (n) | Surviving probability (%) | Deaths  (n) | Surviving probability (%) |  |
| All ages | | | | | | | | | | | |
| 5 | 39 | 95.6% | 8 | 97.2% | 7 | 88.9% | 1 | 99.3% | 23 | 94.0% | 0.001 |
| 10 | 70 | 90.9% | 13 | 94.7% | 7 | 88.9% | 7 | 94.5% | 43 | 86.8% | 0.002 |
| 20 | 109 | 78.1% | 28 | 81.4% | 10 | 76.3% | 13 | 82.0% | 58 | 74.7% | 0.003 |
| 30 | 127 | 61.3% | 39 | 61.1% | 10 | 76.3% | 13 | 82.0% | 65 | 45.6% | 0.002 |
| 40 | 133 | 44.4% | 41 | 54.1% | 10 | 76.3% | 14 | 54.7% | 68 | 11.4% | 0.001 |
| <45 years of age at diagnosis | | | | | | | | | | | |
| 5 | 5 | 98.7% | 4 | 97.3% | 0 | 100% | 1 | 99.0% | 0 | 100% | 0.2 |
| 10 | 7 | 98.1% | 4 | 97.3% | 0 | 100% | 1 | 99.0% | 2 | 97.5% | 0.6 |
| 20 | 15 | 92.6% | 8 | 90.7% | 3 | 84.3% | 2 | 96.5% | 2 | 97.5% | 0.3 |
| 30 | 23 | 81.1% | 13 | 78.4% | 3 | 84.3% | 2 | 96.5% | 5 | 69.5% | 0.4 |
| 40 | 28 | 61.0% | 15 | 69.4% | 3 | 84.3% | 3 | 64.3% | 7 | 23.2% | 0.4 |
| >45 years of age at diagnosis | | | | | | | | | | | |
| 5 | 34 | 93.0% | 4 | 97.0% | 7 | 74.7% | 0 | 100% | 23 | 91.5% | 0.0001 |
| 10 | 63 | 84.5% | 9 | 91.4% | 7 | 74.7% | 6 | 84.6% | 41 | 82.3% | 0.006 |
| 20 | 94 | 61.3% | 20 | 65.4% | 7 | 74.7% | 11 | 45.1% | 56 | 64.2% | 0.1 |
| 30 | 104 | 14.5% | 26 | 0% | 7 | 74.7% | 11 | 45.1% | 60 | 23.3% | 0.3 |
| 40 | 105 | 0% | 26 | 0% | 7 | 74.7% | 11 | 45.1% | 61 | 0% | 0.3 |
